# Effect of Succinamic Acid Derivative on the Growth Kinetics and Induction of Apoptosis in a Cancer Cell Line

**DOI:** 10.7759/cureus.46984

**Published:** 2023-10-13

**Authors:** Manushi Siddharth, Nikhil Khurana, Seema Patel, Suman B Sharma

**Affiliations:** 1 Biochemistry, Multi-disciplinary Research Unit (MRU) at University College of Medical Sciences (UCMS), New Delhi, IND; 2 Biochemistry, University College of Medical Sciences (UCMS), New Delhi, IND; 3 Biochemistry, Employees State Insurance Corporation (ESIC) Medical College and Hospital, Faridabad, IND; 4 Biochemistry, Lady Hardinge Medical College, New Delhi, IND

**Keywords:** alpha hydroxy succinamic acid (α-hsa), head and neck cancer, smokeless tobacco, apoptosis, eugenia jambolana

## Abstract

Introduction

Head and neck cancers are heterogeneous malignancies associated with significant morbidity. Oral cancers are related to the use of tobacco products. Smokeless tobacco usage is a health problem worldwide, and its carcinogenic mechanism is largely unknown. Despite advances in conventional treatments, side effects and drug resistance remain unsolved. Therefore, novel therapeutic agents with minimal side effects using plant derivatives should be explored. An active antihyperglycemic and antioxidant compound known as FIIc was isolated from the fruit pulp of *Eugenia jambolana* (US Patent No.: 2,30,753). Although *E. jambolana* is reported to have anticancer activity, no study has been reported on its growth kinetics and apoptotic potential in the human head and neck cancer cell line (SCC4). The present study evaluated the effect of an herbal compound isolated from the fruit pulp of *E. jambolana* and chemically synthesized the same compound, α-hydroxy succinamic acid (α-HSA), on SCC4 proliferation and apoptotic gene expression.

Methods

The SCC4 cell line was cultured in Dulbecco's Modified Eagle Medium (DMEM). The dosages of smokeless tobacco extract (STE), herbal compound, and synthetic compound were determined by cell viability assay, and their effect on mRNA expression of apoptotic genes was measured by real-time polymerase chain reaction.

Results

The present study observed significant therapeutic effects of the natural and synthetic compounds from the fruit pulp of *E. jambolana *at the concentration range of 100-200 μg/mL on the SCC4 cell line. α-HSA had antiproliferative action; upregulated apoptotic genes like p53, p21, and Bax; and downregulated anti-apoptotic genes like survivin in the SCC4 cell line.

Conclusion

The therapeutic potential of α-HSA and the putative mechanisms involved may be explored to provide the basis for future therapeutic interventions in oral cancer mediated by smokeless tobacco.

## Introduction

Head and neck cancers are complex group of malignancies affecting the oral cavity, nasopharynx, oropharynx, hypopharynx, larynx, paranasal sinuses, and salivary glands. Despite advances in surgery and radiotherapy head and neck cancers are still associated with morbidity and poor prognosis. Squamous cell carcinomas (HNSCC) constitute 90% of all head and neck cancers [1]. Oral squamous cell carcinoma (OSCC) encompasses greater than 90% of all the malignant neoplasms of the oral cavity. In India, it accounts for the third most common cancer in both males and females but the most common cancer among males. It ranks third among the various cancer types contributing to morbidity and mortality in India [2].

Human papillomavirus infection has been subjected as a risk factor for head and neck cancers. Tobacco, betel quid, areca nut, and human papillomavirus are risk factors implicated in the etiopathogenesis of HNSCC [3]. Of all the oral cancers, 95% are related to the use of tobacco products. Smokeless tobacco (ST) products may be consumed orally (moist snuff) or nasally (dry snuff) without burning the product or by chewing/sucking tobacco. ST has been used over centuries by large numbers globally. ST product consumption has been popular in Africa, northern Europe, the United States of America, and several Asian countries, including India and Pakistan. Previous studies have reported that ST contains more than 30 carcinogenic compounds such as tobacco-specific nitrosamines (TSNAs), polycyclic aromatic hydrocarbons, and formaldehyde predisposing to an increased risk of oral, oropharyngeal, and esophageal cancer [4].

Conventional treatments such as surgery, radiotherapy, and chemotherapy are associated with side effects like opportunistic infection and drug resistance. Therefore, it emphasizes the imminent need to develop novel treatment modalities with minimal side effects potentially by using plant derivatives. In recent years, many phytochemicals with low toxicity and anticancer activities have been deciphered. Thus, phytochemicals may represent potential alternative medicine to conventional cytotoxic chemotherapy [5,6].

Herbal medicines are still a potential source of medicine due to the poor accessibility of medical facilities in many remote parts of the world. Herbal medicines have been reported in the scientific literature to possess numerous beneficial biological activities. *E. jambolana *is one such plant that exhibits antidiabetic, anti-atherosclerotic, anti-arthritic, anticancer, antimicrobial, antioxidant, antidiarrheal, and analgesic properties [7,8].

The antihyperglycemic potential of *E. jambolana* has been well explored. Sharma et al. have isolated the active antihyperglycemic compound (FIIc) from the fruit pulp of *E. jambolana *and granted a product patent (No.: 2,30,753) [8]. The chemical structure of herbal compound (FIIc) has been elucidated (α-hydroxy succinamic acid, α-HSA) by spectral analysis. Despite the growing body of data on the chemopreventive potential of edible berry extracts, limited data is available for *E. jambolana* [8].

Based on our current knowledge, scarce information is available regarding the effect of succinamic acid derivative (α-HSA) derived from the *E. jambolana* fruit pulp on the growth kinetics and apoptotic role (level of expression anti-apoptotic and pro-apoptotic protein) in human head and neck cancer cell line (SCC4).

ST usage is a bourgeoning public health problem worldwide. Exposure to ST is carcinogenic to humans. A better understanding of the distinct mechanisms by which tobacco induces carcinogenesis may guide better management and prevention of tobacco-related cancers [9]. The present study examined the therapeutic effect of herbal and chemically synthesized α-HSA isolated from the fruit pulp of *E. jambolana* on the SCC4 cell line. It also evaluated the effect of ST on SCC4 cell proliferation and the effect on the expression level of known anti-apoptotic (BcL-2, survivin) and pro-apoptotic genes (TP53, TP21, and Bax).

## Materials and methods

Smokeless tobacco extract

A commonly used brand of cured tobacco (Khaini) was purchased from the local market; then, 25 g of tobacco was finely chopped, and homogenization was done in distilled water. The mixture was stirred on a magnetic stirrer for 2 hours and kept for 24 hours at 37°C. Thereafter, the supernatant was collected after centrifugation at 5000 g for 20 minutes. Sterilization of the extract was done by passing it through a 0.22 mm filter and stored at 4°C until use [10].

Culture of SCC4 cell line

**Figure 1:**
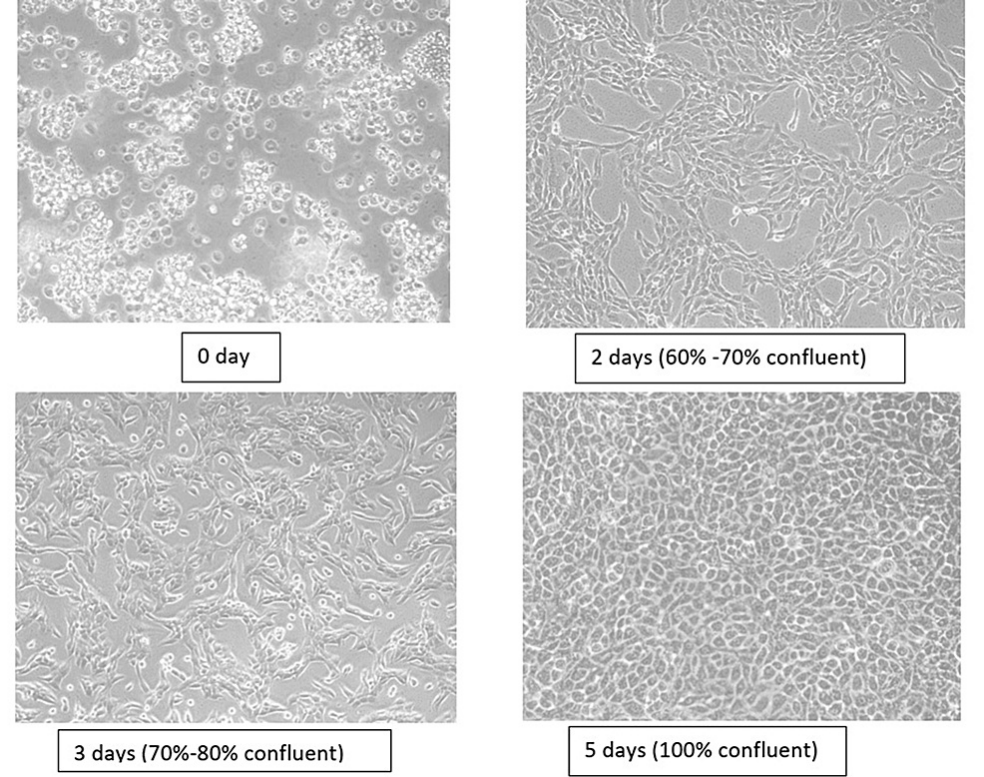
Change of morphology of cells under the inverted microscope

The SCC4 cell line was cultured in DMEM supplemented with 10% fetal bovine serum (FBS), antibiotics, and antimycotic (100 U/mL penicillin, 100 µg/ml streptomycin, 250 µg/ml amphoteric B) in CO_2_ incubator in 5% CO_2_ concentration at 37°C. Media were changed at regular intervals of time. We observed that cells were confluent after seven days of passaging. Cells were checked under the inverted microscope for the change in morphology (Figure 1).

Treatment

For experiments, SCC4 cells were seeded onto 96-well and six-well culture plates. Cells were incubated for 48 hours at 37°C with 5% CO_2_ in 95% humidified air. The medium was replaced with the fresh medium before the experimental procedure. It was followed by treatment with various concentrations of STE (0-1000 µg/ml) for 24 hours. For apoptosis analysis, STE 10 µg/ml was added 24 hours before the co-incubation with an herbal/synthetic compound of HSA. The control cells were treated with prewarmed culture media. Further experimental analysis was continued with extracted cells.

Cell viability assay

Cell viability was measured by assessing the reduction of MTT (3-(4,5-dimethylthiazol-2-yl)-2,5-diphenyltetrazolium bromide) to formazan by the mitochondrial enzyme, succinate dehydrogenase. Cells were seeded onto 96-well plates (4.5 x 104 cells) maintained at 37°C in 5% CO_2_ for 48 hours, until 80% confluent. STE, synthetic, and herbal compounds were then added at varying concentrations (0-1000 µg/ml) in the medium for 24 hours accordingly. A negative control was included, which consisted of cells incubated with the media exclusively. After treatment for 24 hours, cells were washed with phosphate buffer saline (PBS) followed by the addition of 25 µl of MTT (5 mg/mL) in PBS to 225 µl of fresh medium in each well. After incubating for three hours at 37°C, the supernatant was removed, the insoluble formazan crystals were dissolved in 100 µl of dimethyl sulfoxide (DMSO), and the absorbance was determined at 450 nm using a multimode reader, SpectraMax M2e (Molecular Devices, Sunnyvale, CA). The results were expressed as the percentage (%) of viability with respect to control (untreated cells). The method was previously standardized [11].

Quantitative real-time PCR

Total RNA extracted from the treated and untreated cells was treated with the TRIzol (TRI) reagent (Invitrogen, Carlsbad, CA). cDNA was synthesized by taking 1 µg of total RNA using the Maxima First Strand cDNA Synthesis Kit (Thermo Fisher Scientific, USA). Quantitative real-time PCR (LC480, Roche, Basel, Switzerland) was performed to measure the expression of mRNA for the target gene using SYTO 9 fluorescent dye. The reaction mixture contained template cDNA, maxima master mix (Thermo Fisher Scientific, USA), and 10 pmol of each primer of both the target gene and housekeeping gene (18S). PCR was performed consisting of 35 cycles each constituting denaturation for 1 s, annealing at 54ºC-58ºC for 10 s, and final extension for 10 s. Further, relative or fold change in expression level was calculated by 2 - (ΔΔ Ct). The protocol was standardized previously [12].

Statistical analysis

All treatment data were normalized to non-treated controls. Data are expressed as mean ± standard error of the mean (SEM) from three or more independent experiments. Comparison between different groups was performed by one-way analysis of variance (ANOVA), followed by post-hoc comparison with Sidak’s test. In all tests, the criterion for statistical significance was p < 0.05.

## Results

**Figure 2:**
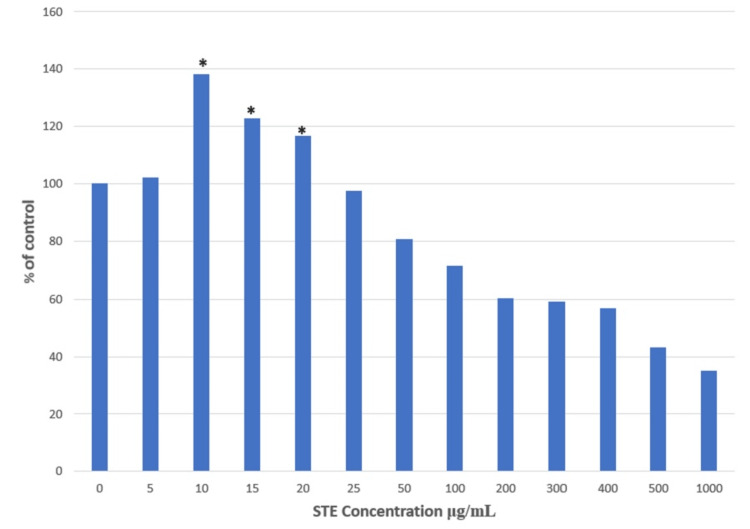
Measurement of cell viability in SCC4 cells treated with STE by MTT assay Significant at p < 0.05. * represents significance compared to the non-treated control group. SCC4: Human head and neck squamous cell carcinoma cell line; STE: Smokeless tobacco extract; MTT: 3-(4,5-dimethylthiazol-2-yl)-2,5-diphenyltetrazolium bromide.

The cells were confluent after seven days of passaging, and the cells were checked under the inverted microscope for the change of morphology (Figure 1). The optimized dose of STE was evaluated using MTT assay, where it was observed to significantly proliferate the SCC4 cell line culture. The cells were treated with STE in the concentration range of 0-1000 μg/mL for 24 hours, and an MTT assay was performed. Significantly increased cell viability was observed in the range of 10-20 μg/mL with a maximum concentration of 10 μg/mL. The cell viability was observed to decline significantly at the higher concentration range of 25-1000 μg/mL. Therefore, a concentration of 10 μg/mL was used for further experiments (Figure 2).

**Figure 3:**
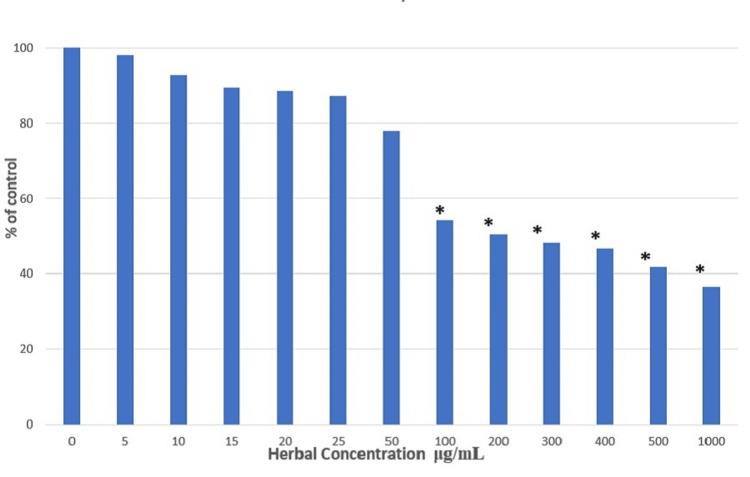
Measurement of cell viability in SCC4 cells treated with herbal compound by MTT assay Significant at p < 0.05. * represents significance compared to the non-treated control group. SCC4: Human head and neck squamous cell carcinoma cell line; MTT: 3-(4,5-dimethylthiazol-2-yl)-2,5-diphenyltetrazolium bromide.

The ameliorating effect of the herbal compound was evaluated in the STE-induced SCC4 cell line. The cells were treated with various concentration ranges of 0-1000 μg/mL for 24 hours. STE previously induced the cells for 24 hours. The cell viability seemed to decrease at the concentration of 50 μg/mL, whereas LC50 of the herbal compound was observed at 200 μg/mL. Therefore, two concentrations, 100 and 200 μg/mL, were used for further experiments (Figure 3).

**Figure 4:**
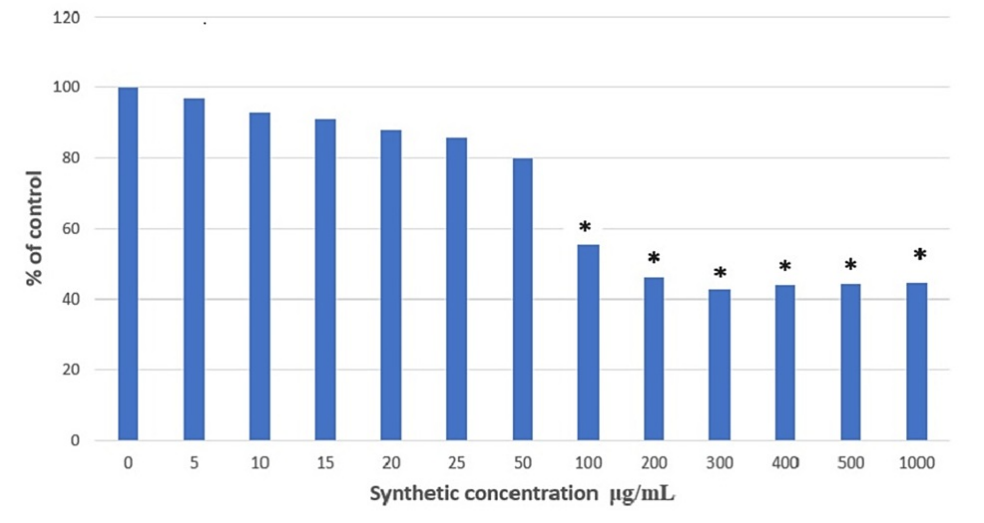
Measurement of cell viability in SCC4 cells treated with synthetic compound (α-HSA) by MTT assay Significant at p < 0.05. * represents significance compared to the non-treated control group. SCC4: Human head and neck squamous cell carcinoma cell line; MTT: 3-(4,5-dimethylthiazol-2-yl)-2,5-diphenyltetrazolium bromide; α-HSA: alpha-hydroxy succinamic acid.

Further, the present study evaluated the ameliorating effect of the synthetic compound (α-HSA) in the STE-induced SCC4 cell line. The cells were treated with various concentrations ranging from 0 to 1000 μg/mL for 24 hours. The cells had previously been induced for 24 hours by STE. The cell viability appeared to decrease at the concentration of 50 μg/mL, whereas LC50 of α-HSA synthetic compound was observed between 100 and 200 μg/mL. Therefore, two concentrations, 100 and 200 μg/mL were used for further experiments (Figure 4).

**Figure 5:**
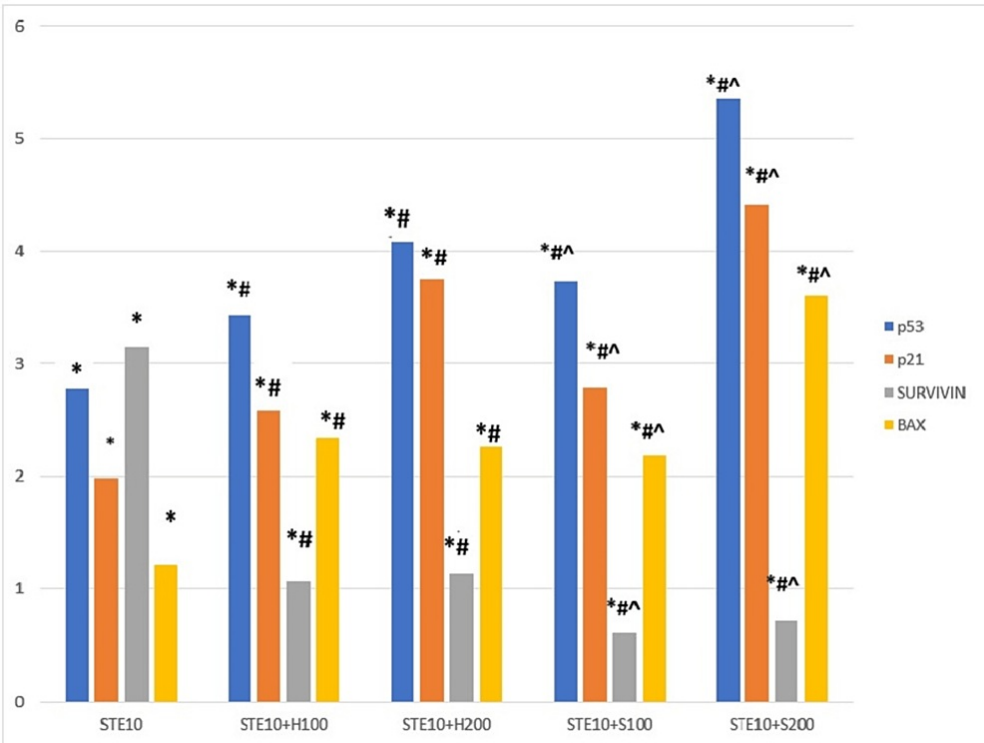
α-HSA on mRNA expression of different apoptotic gene marker Significant at p < 0.05. * represents significance compared to the non-treated control group. # represents significance compared to the STE-treated group. ^ represents significance compared to the herbal compound-treated group. α-HSA: Alpha-hydroxy succinamic acid; STE: Smokeless tobacco extract.

The apoptotic gene mRNA expression, such as p53, p21, Bax, and survivin, was observed in the treated cells. The mRNA expression of p53, p21, and Bax genes was observed to be significantly upregulated in all the treated groups, ranging from two- to five-fold in comparison to non-treated cells. On the other hand, survivin gene expression showed significant downregulation in all treated groups except the STE group. When the results were compared between the STE-treated group and the cotreated group with herbal and synthetic HSA, the mRNA expression of p53, p21, and Bax genes showed a significantly increased fold change range of up to 2.5-5.3 in the herbal and synthetic compound-treated group. In contrast, the survivin gene showed significant downregulation in these groups. If we compared the herbal and synthetic compounds, we observed an increased fold change range of up to 2.2 to 3.2 in the synthetic α-HSA compound-treated group, whereas the survivin gene had a decreased fold change (Figure 5).

## Discussion

The actual burden of head and neck cancer in India is much larger than reflected in the existing literature. Late diagnosis, resistance to conventional therapy, metastasis, and relapse lead to poor five-year survival rates of patients with HNSCC. Most head and neck cancers are squamous cell carcinomas that develop in the upper aerodigestive epithelium after exposure to carcinogens such as tobacco and alcohol [13]. Exposure to ST consumed orally/nasally is a carcinogenic and an ever-increasing problem for humans [4]. Initiation of tobacco use gradually leads individuals to become addicted to nicotine. ST contains more than 30 carcinogens, especially a greater number of tobacco-specific nitrosamines such as 4-(methylnitrosamino)-1-(3-pyridyl)-1-butanone (NNK) and N'-nitrosonornicotine (NNN). These carcinogens are taken up, distributed, metabolized, and detoxified and may lead to DNA adduct formation. DNA adducts once formed may lead to sister chromatid exchanges, chromosomal aberrations, micronuclei, and neoplastic changes [14]. If the DNA adducts formed persist, they evade the normal cellular repair systems, leading to permanent DNA mutations. Mutations in the RAS oncogene or the P53 tumor suppressor gene result in the loss of normal healthy cellular growth control, progressing to neoplastic cell proliferation and cancer [15,16]. Besides, disruption of harmony between pro- and antioxidant mechanisms due to oxidative stress, reactive oxygen species, and chronic local inflammation caused due to ST may promote an ideal microenvironment promoting tumor [17].

Almost 80% of the world's population endorses the use of herbal drugs for the treatment of various diseases. Certain foods contain bioactive compounds with potential chemopreventive properties; encouraging the consumption of such foods may demonstrate a valuable strategy in cancer prevention. *E. jambolana*, commonly known as Jamun or Indian Blackberry, is a fruit-bearing tree native to the Indian subcontinent and is found in various tropical regions around the world [18]. It has a long history of use in traditional Indian medicine (Ayurveda) due to its diverse chemical constituents and potential medicinal properties [19,20].

The antiproliferative and pro-apoptotic effects of *E. jambolana* fruit extract (JPE) have been documented in estrogen-dependent/aromatase-positive and estrogen-independent breast cancer cells and colon cancer cells [21-25]. However, research on the pro-apoptotic and anticancer properties of *Jambolana *extract on HSCC is relatively scarce.

The active antihyperglycemic compound known as alpha-hydroxy-succinamic acid (FIIc) (US patent number: 6,426,826 dated 6th August 2002; Indian product patent number.: 2,30, 753 dated February 2009) from the fruit pulp of *E. jambolana *has already been isolated by Sharma et al. [8]. Therefore, succinamic acid derivatives are expected to possess antidiabetic and antioxidant properties [26].

The results from the present study showed the significant effect of ST on SCC4 cell proliferation or cell metastasis at the concentration of 10 μg/mL. This study also reported the therapeutic effect of natural and synthetic compounds (α-HSA) isolated from *E. jambolana* at the concentration of 100-200 μg/mL on the SCC4 cell line. Moreover, the synthetic compound of HSA showed more notable results at the concentration of 200 μg/mL. α-HSA had antiproliferative action and could upregulate apoptotic genes like p53, p21, and Bax and downregulate anti-apoptotic genes like survivin in the SCC4 cell line.

Thus, it reveals the anticancer potential of α-HSA to mitigate the effect of ST-induced cancer by intervening in its carcinogenicity mechanism. Moreover, the antihyperglycemic and antioxidant activity of α-HSA has been reported in α-HSA-treated rats, which showed marked improvement in glycemic markers and total antioxidant capacity (TAC) in comparison to diabetic control rats [26].

Hence, by modulating apoptosis and antioxidant potential, α-HSA may provide a basis for therapeutic intervention in combating the carcinogenic potential of STE. In a vast and diverse nation like India, there exists a substantial disparity in the accessibility of healthcare infrastructure and specialized expertise for the treatment of head-neck cancer patients. Furthermore, uniform adherence to evidence-based practices in managing this cancer is lacking. Consequently, a pressing need to establish a holistic approach to the prevention and treatment of cancer is the need of the hour. Researchers should aim to elucidate the precise mechanisms through which dietary phytochemicals exert their anticancer effects while minimizing undesirable side effects.

Limitations

The apoptotic pathway was not completely studied covering only some major apoptotic genes. In vivo studies (animal models) were not performed. Correlation between oxidative stress markers generated by ST which contribute to tumor microenvironment could be performed.

## Conclusions

α-HSA had antiproliferative action and could upregulate pro-apoptotic genes like p53, p21, and Bax and downregulate anti-apoptotic genes like survivin in the SCC4 cell line. The promising results of α-HSA warrant future studies to provide valuable mechanistic insights into how it exerts anticancer effects. This study indicated the putative mechanisms involved, which may eventually provide the basis for future therapeutic interventions in squamous cell epithelial cancer mediated by ST.
